# Accuracy of automatic syndromic classification of coded emergency department diagnoses in identifying mental health-related presentations for public health surveillance

**DOI:** 10.1186/1472-6947-14-84

**Published:** 2014-09-23

**Authors:** Henning TG Liljeqvist, David Muscatello, Grant Sara, Michael Dinh, Glenda L Lawrence

**Affiliations:** 1 NSW Public Health Officer Training Program, New South Wales Ministry of Health, Sydney, NSW, Australia; 2 School of Public Health and Community Medicine, University of New South Wales, Sydney, NSW, Australia; 3 Mental Health and Drug and Alcohol Office, NSW Ministry of Health, Sydney, NSW, Australia; 4 Trauma Services, Royal Prince Alfred Hospital, Sydney, NSW, Australia; 5 Discipline of Psychiatry, Sydney Medical School, University of Sydney, Sydney, NSW, Australia; 6 Sydney Medical School, University of Sydney, Sydney, NSW, Australia

## Abstract

**Background:**

Syndromic surveillance in emergency departments (EDs) may be used to deliver early warnings of increases in disease activity, to provide situational awareness during events of public health significance, to supplement other information on trends in acute disease and injury, and to support the development and monitoring of prevention or response strategies. Changes in mental health related ED presentations may be relevant to these goals, provided they can be identified accurately and efficiently. This study aimed to measure the accuracy of using diagnostic codes in electronic ED presentation records to identify mental health-related visits.

**Methods:**

We selected a random sample of 500 records from a total of 1,815,588 ED electronic presentation records from 59 NSW public hospitals during 2010. ED diagnoses were recorded using any of ICD-9, ICD-10 or SNOMED CT classifications. Three clinicians, blinded to the automatically generated syndromic grouping and each other’s classification, reviewed the triage notes and classified each of the 500 visits as mental health-related or not. A “mental health problem presentation” for the purposes of this study was defined as any ED presentation where either a mental disorder or a mental health problem was the reason for the ED visit. The combined clinicians’ assessment of the records was used as reference standard to measure the sensitivity, specificity, and positive and negative predictive values of the automatic classification of coded emergency department diagnoses. Agreement between the reference standard and the automated coded classification was estimated using the Kappa statistic.

**Results:**

Agreement between clinician’s classification and automated coded classification was substantial (Kappa = 0.73. 95% CI: 0.58 - 0.87). The automatic syndromic grouping of coded ED diagnoses for mental health-related visits was found to be moderately sensitive (68% 95% CI: 46%-84%) and highly specific at 99% (95% CI: 98%-99.7%) when compared with the reference standard in identifying mental health related ED visits. Positive predictive value was 81% (95% CI: 0.57 – 0.94) and negative predictive value was 98% (95% CI: 0.97-0.99).

**Conclusions:**

Mental health presentations identified using diagnoses coded with various classifications in electronic ED presentation records offers sufficient accuracy for application in near real-time syndromic surveillance.

## Background

Syndromic surveillance in Emergency Departments (EDs) endeavours to achieve near real-time monitoring of routinely collected health-related data to signal uncommonly high levels of presentations of particular syndromes [1-3]. Syndromes may be collections of symptoms or characteristics which are grouped into categories, often focussed on early pre-diagnostic signs and symptoms, or prodromes. Syndromic surveillance has proven useful in the early detection of trends in emergency department presentations for a variety of syndromes [4-6]. One of the challenges in designing a surveillance system is to achieve the best balance between sensitivity and specificity. An over-sensitive system is likely to produce false positives and false alarms, and to be less specific than is useful, while the opposite may result in missing important signals. Good stewardship of human and other resources demands efforts to achieve the optimal balance based on available resources and technology.

Measurements of the accuracy of ED data streams to identify particular syndromes using International Classification of Diseases (ICD) and Systematized Nomenclature of Medicine - Clinical Terms (SNOMED CT) concepts [7,8] have been carried out before [9,10], but the syndrome of mental health-related ED presentations lacks such attention.

Inclusion of “Mental Health” as a syndrome in routine real-time surveillance is considered appropriate for a number of reasons. Mental illness accounts for a greater disability burden in developed countries than any other disease group, including cancer and heart disease [11]. Monitoring ED presentations related to mental health problems is important as it provides information relevant to policy, planning and guidance for interventions that may be required locally or on a broader scale [12].

In addition, increases in mental health-related ED presentations may occur due to emergencies or disasters causing collective anxiety. Natural and man-made disasters may disrupt access to mental health services and medications, which could result in an increase in both the number and severity of ED presentations for mental health problems. One of the threats that led to the relatively recent introduction of, and surge in, syndromic surveillance was the potential use of chemical, biological and radiological (CBR) weapons by governments or terrorists [13-15]. Some of these agents can cause alterations in mental health state on a mass scale. Potentially, industrial accidents may result in release of toxins or chemicals with mind altering effects.

Development of methods to rapidly collect disease data for this purpose has accelerated in recent years. Greater use of electronic medical records and patient management information systems has improved the completeness and uniformity of data collection systems [16,17]. These developments may have improved the accuracy of ED syndromic surveillance.

In New South Wales (NSW), syndromic surveillance in EDs was established in 2003 [5,9]. Automated syndromic surveillance operates in 59 EDs across the state and provides daily monitoring of ED visits presenting with various health problems grouped into 38 syndromes. Syndrome groupings are allocated according to the provisional ED diagnosis which is the first recorded diagnosis and is a mandatory field. The 59 EDs accounted for approximately 84% of all ED activity in NSW, and includes almost every major city ED and one or more of the largest EDs in each rural administrative area. The number of presentations in each syndrome is monitored over time to identify unusual patterns of illness, which could indicate an emerging outbreak of disease. One syndrome monitored is labelled “Mental health problems”. It includes diagnoses for mental health conditions and problems including hallucinations, nervousness, restlessness, hostility, suicidal ideation, and other emotional and behavioural symptoms (Table 1). Drug and alcohol intoxication are excluded because they are included in alternative syndromes. Depending on their patient administration system, EDs participating in routine surveillance report ED diagnoses using any of the ICD-9, ICD-10 and SNOMED CT concepts (Additional file 1). The diagnoses are selected by ED clinicians in the ED records at patient discharge or admission to a hospital ward.

**Table 1:** Mental health codes in ICD 9 and ICD 10 and SNOMED CT concepts

***Coding system***	***Codes***
ICD-9	290, 293.2-302, 306–307.80, 307.82-316.99, 799.2, V71.0
ICD-10	F03-F04, F06-F09, F20-F54, F59-F69, F84-F99, R44-R46, Z03.2, Z04.6, Z86.5
SNOMED CT	SNOMED CT concepts that map to any of the ICD-9 or ICD-10 codes (For complete list of concepts, see Additional file 1)

Syndromic surveillance relies on accurate recording of information in the data sources that are used. The aim of this study was to estimate the accuracy of using emergency department coding to identify mental health related visits. To our knowledge, there have been no studies investigating the accuracy of mental health codes compared with written information from triage nurse notes.

## Methods

### Sampling

A random sample of n = 500 electronic records was selected from a database of all recorded ED visits (N = 1,815,588) in calendar year 2010 for the 59 hospitals participating in ED syndromic surveillance. In choosing the sample size, we balanced the burden of manual classification by the clinicians and the need for reasonably constrained confidence intervals.

### Identifying mental health related ED visits

For the study, the sample of 500 records was classified in two ways. First using the automated syndrome grouping based on the diagnosis code for “Mental health problems” and second using a reference standard. Bertens, et al. [18] described criteria for external validation of reference standards when using expert panels in diagnostic research. Their recommendations include four considerations: constitution of the expert panel; information presented to experts; decision process (e.g. classification or case definition); and validity of the expert’s diagnoses (e.g. agreement testing). We followed these criteria when establishing the reference standard in this study.

The reference standard was obtained using the triage nurse notes included on the surveillance records. The notes were examined separately by three clinicians: a psychiatrist (Psych), an ED physician (EDP) and an Intensive Care Paramedic (pre-hospital emergency clinician (PHEC)). These three practice areas were selected in order to represent three phases of the patient journey and hence three different clinical perspectives. Each clinician was blinded to the diagnostic code recorded in the electronic record and to the assessment of the other two clinicians.

From the triage notes, the clinicians determined whether or not each of the 500 sampled presentations was due to a mental health condition according to the study case definition (see Table 2). Presentations were counted as mental health-related visits if the clinician regarded mental health reasons as being either the primary complaint, or a major contributing cause for the complaint leading to the visit.

**Table 2:** Case definitions

***Category***	***Definition***
Mental disorders	Mental disorders are defined according to the clinical diagnostic criteria. For this study, a mental disorder is defined as a condition which affects a person’s cognitive, emotional or social abilities and attracts a diagnosis of psychiatric illness.
Mental health problems	This includes, but is not restricted to, such things as, stress, anxiety or depression. Individuals with mental health problems may never meet the diagnostic threshold for a mental disorder.
Inclusions	-A mental disorder or mental health problem is the main reason for the ED visit.
	-A mental disorder or mental health problem is a major contributing factor leading to the condition which is the main reason for the ED visit.

A “mental health problem presentation” for the purposes of this study is defined as any ED presentation where either a mental disorder or a mental health problem is the reason for the ED visit.

The clinicians’ results were assessed for agreement with each other using the Kappa statistic for inter-rater reliability in SAS [19]. There are several ways of measuring agreement, but the Kappa statistic is used most frequently in the medical literature [20]. Viera and Garrett described a scale for interpreting the Kappa statistic: <0 = Less than chance agreement; 0.01-0.20 = Slight agreement; 0.21-0.40 = Fair agreement; 0.41-0.60 = Moderate agreement; 0.61-0.80 = Substantial agreement; and 0.81-0.99 = Almost perfect agreement [20].

This was done to assess whether the clinicians’ opinions were in agreement as a measure of reliability and to provide a valid reference standard against which to compare the automatic classification of coded ED diagnoses. A reference standard called ‘AllClin’ was created which included the records about which all three clinicians agreed.

After establishment of the ‘AllClin’ reference standard, the automated coding from the ED syndromic data extraction was compared with the reference standard using the Kappa statistic to assess agreement. Finally, the ‘AllClin’ reference standard was used to estimate the sensitivity, specificity and positive predictive value (PPV) and negative predictive value (NPV) of the ED syndrome-coded method of identifying mental health-related visits to EDs. As an additional level of investigation, we examined the triage notes and provisional diagnoses of the false positive and false negative cases.

## Results

From the random sample of 500 records of any-cause ED visits selected from the NSW ED syndromic surveillance database, the code group for mental health identified 21 (4.2% (95% CI 2.4% - 6%)) mental health related presentations.

### Agreement between the clinicians

Agreement between the 3 clinicians in identifying mental health-related presentations by reviewing the written triage notes of the 500 sampled presentations was almost perfect (Kappa 0.81-0.88) see Table 3).

**Table 3:** Clinician’s agreement with each other in identifying mental health related ED visits in the 500 sample records

Psychiatrist compared with emergency department physician			
Kappa score	0.81	P = < 0.001	95% CI (0.70 - 0.92)
Pre-hospital emergency clinician compared with psychiatrist			
Kappa score	0.86	P = < 0.001	95% CI (0.77 - 0.96)
Emergency department physician compared with pre-hospital emergency clinician			
Kappa score	0.88	P = < 0.001	95% CI (0.79- 0.97)

### Agreement between automatic syndromic classification of coded ED data and the reference standard

Agreement between automatic ED surveillance and the ‘AllClin’ reference standard was substantial with Kappa = 0.73 (95% CI: 0.58 - 0.87) (Table 4). Sensitivity of coding for mental health visits against the AllClin reference standard was 68% (95% CI: 0.46 - 0.84) and specificity was 99% (95% CI: 0.98 - 0.997). The positive predictive value was 81% (95% CI: 0.57 - 0.94) while the negative predictive value was 98% (95% CI: 0.97- 0.99). Four cases were grouped by the automated system as being mental health related while picked as negative by the clinicians (false positives) and eight cases were identified by the clinicians but not by the automated system (false negatives). The four cases that were identified as positive by the coded extraction but negative by the clinicians suggest incorrect diagnostic choice in the ED and mistakes in the ED data entry process. The main complaints of these cases were: scared with chest pain, post ictal, dizzy and “other”. The eight false negative presentations included free text information in the triage notes which indicated to the clinicians that a mental health problem or condition was the reason for the visit while the provisional diagnosis was recorded as something else.

**Table 4:** Automatic syndromic classification of coded emergency department diagnoses measured against the reference standard of AllClin*

	**AllClin**		
Coded surveillance^♣^	MH +	MH -	Total
MH +	17	4	21
MH -	8	471	479
Total	25	475	500
Kappa score	0.73	P = < 0.001	95% CI (0.58 - 0.87)
Sensitivity	68%		95% CI (46% - 84%)
Specificity	99%		95% CI (98% - 99.7%)
PPV	81%		95% CI (57% - 94%)
NPV	98%		95% CI (97%- 99%)

*AllClin: Records about which all three clinicians agreed.

^♣^Coded surveillance: Automatic syndromic classification of coded emergency department diagnoses.

## Discussion

We found that the accuracy of using emergency department coding with ICD 9, ICD 10 and SNOMED CT concepts to identify mental health-related visits in EDs was satisfactory. Compared with clinician review of corresponding triage notes the routine system performed with high specificity and lower but still acceptable sensitivity as it identified over two thirds of mental health related visits to EDs.

It is reasonable to assume that specificity must be high since a record must be made of the reason for the visit being within the mental health set of parameters. On the other hand, as suggested by examination of the triage notes of the false negative cases, a mental health condition may be a major contributing reason for an ED visit while not being the immediate condition treated. This would not be possible to identify by means of ED coding, and thus would reduce sensitivity. Despite this, the ED surveillance system assessed in this study provided sensitive results. Due to the accuracy of automatic syndromic classification of coded emergency department diagnoses in identifying mental health-related presentations, it is justified to continue to include it in routine public health surveillance.

This study was confined to the use of ED triage notes and ED diagnostic codes partly for logistical reasons, but also for the purpose of assessing this early, often pre-definitive diagnosis phase of the patient journey in an attempt to promote early detection of changes in patterns in mental health-related visits to EDs. Our study provides a foundation for future studies of trends and time series analyses in mental health presentations to EDs.

The current study examined the internal consistency of diagnosis within a clinical record and did not have an external diagnostic reference standard. However, as recommended by Bertens, et al. [18] we used three separate areas of clinical expertise, presented the information in a uniform manner with each clinician blinded to the result of the others, provided a clear case definition of mental health-related problems, and finally tested the agreement between the clinicians. Therefore, we believe that the reference standard was relevant to the aim of the study, which was to assess the utility of diagnostic codes for routine syndromic surveillance of mental health.

Another potential limitation of this study, but at the same time one of the motivations for it, is that clinical staff, sometimes experiencing competing priorities, must enter the coded data in patient records. This also occurs in different settings, e.g. rural, city, day-time, night-time, busy and less busy times and at different facilities between which there may be variations. This study cannot quantify the impact of such potential inconsistencies. The presence of the false positive cases, however, indicates that there may be mistakes in the data entry process. The inconsistencies we identified were due to provisional diagnoses labelling a mental health problem, such as anxiety when further examination of the record demonstrated that the problem was somatic, for example cardiac.

Syndromic surveillance by automatic syndrome grouping of coded ED diagnoses has been assessed previously by Liljeqvist et al. [9] in a study of influenza-like illness (ILI), which found that grouped coding was both sensitive and specific for the identification of ILI for ED visits. The mental health syndrome grouping is considerably broader in scope than that of ILI and thus may offer greater challenges in syndrome grouping. On the other hand, accuracy might decline if we were attempting to identify more specific mental health syndromes such as “anxiety”.

Skovgaard et al. [10] measured the accuracy of ICD-10 codes in the diagnostic classification of mental health problems in children aged 1½ years. Their study involved a smaller sample than our study and included a detailed examination by clinical specialists. They found that ICD-10 codes offer a sufficient frame for classification of mental health conditions in 1½ year old children if used by highly specialised clinicians in controlled settings.

Inconsistencies between triage notes and discharge records may demonstrate that a person’s initial complaint was not the actual cause of the problem leading to the ED visit. Fleischauer and others [21] used Kappa statistics to assess agreement between syndromic grouping of the chief complaint on presentation in EDs with discharge diagnoses in patient records. They found moderate agreement overall and recommended that the validity of automated surveillance coding could be improved by including discharge diagnoses. The clinical examination of triage notes performed in our study demonstrates an option which may offer more timely results and more sensitive identification than automated syndromic groupings alone and thus could identify cases which would otherwise be missed by automated methods.

Some challenges in ED surveillance have been described by authors such as Gorelick et al. [22] who discussed the problems inherent with coding within EDs and suggested that further study is required into coding practices in the ED setting. The high degree of unpredictability in the ED environment and varying degrees of competence among staff in coding, they suggest, threaten validity of coded data automatically extracted from EDs. The same argument is provided by O’Malley and others [23] who explored actual coding practices. They found that errors were made due to issues such as patient-clinician communication problems, lack of training in coding among coders, large amounts of information with lack of attention to detail by clinicians and both unintentional and intentional miscoding.

In 2000, Hirshon [24] described how limitations to ED data collection occurred due to lack of uniformity in data collection and in data entry. While some of these issues have been addressed through increased use of electronic systems and increased standardisation of medical records internationally, they are still improving and require further assessment.

Deliberations about any syndromic surveillance system that collects data from EDs should consider the large number of diagnoses that can be recorded and the lack of coding rules in ED triage compared with, for example, the coding in formal post-hospital admission records, such as discharge or separation records, which would offer more careful and accurate diagnostic data than the ED setting. There must be a trade-off between accuracy and timeliness however. This study identifies that there is a level of accuracy in ED mental health syndromic coding which makes it a useful addition to comprehensive surveillance despite the complexities within which it operates and while providing near real-time results.

Considering that increases in, or changes to, mental health-related ED presentation patterns can be triggered by a large range of events and community needs, achieving comprehensive surveillance that can identify changes in patterns as early as practicable is a high priority.

Another reason for including mental health problems in coded syndromic ED surveillance is the high burden mental health-related visits place on EDs. While the state of mental health of the population may not traditionally have been something for which early warning was a key priority, rather monitoring of trends over time was the goal, mental health has increasingly become a focus of emergency response organisations internationally and in Australia [12,25]. Continuing to monitor mental health as a syndrome as is currently conducted in NSW offers a sufficiently sensitive gauge to detect mental health related visits in EDs. Increased sensitivity may be desired under some circumstances and then a practical solution, at least on a local level, would be to examine the text in triage notes as we demonstrated.

Syndromic surveillance cannot in itself offer a comprehensive epidemiological picture of any disease, but provides an important component of the most comprehensive surveillance programs. One of the weaknesses of syndromic surveillance is its lack of sensitivity and specificity in identifying specific diseases. As it monitors syndromes such as influenza like illness, chest pain or fever, it can only be an indicator of disease trends. A broad “mental health” syndrome is possibly the most challenging to define as it covers a vast range of conditions, some diagnosable and others not, some presenting as the actual reason for an ED visit and some hidden behind other conditions such as physical trauma or cardiac disease.

## Conclusions

This study demonstrates that grouping of diagnosis codes from ED information systems for syndromic surveillance using ICD 9, ICD 10 and SNOMED CT concepts can identify mental health visits in EDs, and that it can identify these visits with adequate sensitivity and specificity to be useful in routine rapid syndromic surveillance.

Under circumstances where either automated signals indicate an anomaly or known events warrant deeper investigation, it may be useful to use clinicians to examine the ED records in more detail. Further development of free text extraction such as explored by Travers, et al. [26] may also be useful in increasing sensitivity of syndromic surveillance of mental health related visits in EDs. Ongoing analyses of the accuracy of syndromic ED coding of mental health related visits would be useful in increasing the depth of understanding of the available data and of the epidemiology of mental health conditions.

### Ethical approval

Ethical approval was provided by New South Wales Population Health and Health Services Research Ethics Committee: LNR/11/CIPHS/71.

### Consent

The source data for this study were from patient information systems used routinely in New South Wales public hospitals and reported to the NSW Ministry of Health for public health surveillance purposes. The source data available for the study did not include database fields for patient name, date of birth or address so obtaining informed consent was not feasible. The study protocol was approved by the New South Wales Population Health and Health Services Research Ethics Committee.

## Competing interests

The authors declare that they have no competing interests.

## Authors’ contributions

HL was the lead author, he designed the study, managed the project, collected and analysed the data, and wrote the manuscript and provided clinical analysis of the triage data. DM advised in study design and statistical analysis, he also carried out critical revisions of the manuscript. MD and GS provided clinical analysis of the triage data and editorial advice, GS also revised the manuscript. GL contributed to the analysis and interpretation of the data and critically revised the manuscript for intellectual content and gave editorial advice. All authors approved the final version of the manuscript.

## Pre-publication history

The pre-publication history for this paper can be accessed here:

http://www.biomedcentral.com/1472-6947/14/84/prepub

## Supplementary Material

Additional file 1 List of SNOMED CT concepts. Click here for file
